# The MCM2-7 Complex: Roles beyond DNA Unwinding

**DOI:** 10.3390/biology13040258

**Published:** 2024-04-13

**Authors:** Brooke D. Rankin, Susannah Rankin

**Affiliations:** 1 Cell Cycle and Cancer Biology Program, Oklahoma Medical Research Foundation, Oklahoma City, OK 73104, USA; brooke-rankin@omrf.org; 2 Cell Biology Department, University of Oklahoma Health Sciences Center, Oklahoma City, OK 73104, USA

**Keywords:** MCM2-7 complex, origin licensing, transcription-replication conflicts, DNA damage signaling, histone recycling

## Abstract

**Simple Summary:**

Here, we summarize what is known about the non-replication roles of MCM helicase, best known for its ability to unwind double-stranded DNA templates during DNA replication.

**Abstract:**

The MCM2-7 complex is a hexameric protein complex that serves as a DNA helicase. It unwinds the DNA double helix during DNA replication, thereby providing the single-stranded replication template. In recent years, it has become clear that the MCM2-7 complex has additional functions that extend well beyond its role in DNA replication. Through physical and functional interactions with different pathways, it impacts other nuclear events and activities, including folding of the genome, histone inheritance, chromosome segregation, DNA damage sensing and repair, and gene transcription. Collectively, the diverse roles of the MCM2-7 complex suggest it plays a critical role in maintaining genome integrity by integrating the regulation of DNA replication with other pathways in the nucleus.

## 1. Introduction

An ordered series of protein-loading and activation steps ensures that DNA replication occurs efficiently and, importantly, only once per cell cycle. A critical regulated step, referred to as origin licensing, is defined as the loading of the MCM (mini-chromosome maintenance) helicase [1]. This helicase is a hetero-hexamer of ATPase subunits, MCM2-7 [2,3]. The MCM2-7 complex forms a ring-shaped structure that, upon activation, engages the DNA in a manner that promotes unwinding of the DNA double helix, allowing access by polymerases and other components of the active replisome [4,5,6].

Replisome assembly begins with the binding of the Origin Recognition Complex (ORC) to presumptive origins [7,8]. In higher eukaryotes, origins are not sequence-specified as they are in some fungal models. ORC binds throughout the genome as cells exit mitosis, and in vertebrates replication licensing occurs when a replication inhibitor, called geminin, is degraded at mitotic exit [9,10]. This releases the licensing factor, Cdt1, which, together with Cdc6, promotes loading of the MCM2-7 complex onto chromatin [11,12,13].

Throughout G1, MCM2-7 complexes remain bound to chromatin but do not unwind the double-stranded DNA (dsDNA). Replication initiation occurs when MCMs are activated by two cell-cycle-dependent kinases, Dbf4-dependent kinase (DDK) and cyclin-dependent kinase (CDK) [14,15,16,17]. Phosphorylation of MCMs allows association of the helicase-activating components, GINS and Cdc45, that, together with MCM2-7, form the active CMG holo-helicase [18,19]. Upon activation, the helicase translocates from the origin along a single strand of the helix, unwinding DNA in the 3′-5′ direction, thus forming replication “bubbles” as pairs of replication forks are established and progress away from sites of initiation [20].

Upon completion of DNA replication, the replisome is disassembled and the MCM2-7 complex is unloaded. The CMG helicase is removed from the DNA after polyubiquitination of the MCM7 subunit [21]. In vertebrates, this modification is made by the E3 ubiquitin ligase CRL2^LRR1^ (SCF^Dia2^ in yeast) [22,23]. Polyubiquitinated MCM7 is recognized and removed from chromatin by the protein remodeler p97/VCP/Cdc48 [24]. The MCM-binding protein (MCM-BP) and its interacting partner USP7 have also been shown to function in unloading MCM2-7 from chromatin, but it has been suggested that they function primarily on MCM2-7 complexes that remain at inactive origins [25,26,27].

In what has been referred to as the “MCM paradox”, many more MCM2-7 complexes are loaded during replication licensing than are required for efficient DNA replication [28,29]. Some estimates suggest that there is a ten- to twenty-fold superabundance of MCM2-7 complexes on chromatin. But why? Several models have been invoked to explain this phenomenon, and the most prominent proposes that these complexes can be engaged if necessary, should DNA replication be compromised in some way, thereby providing robustness to the system. During replication stress, “dormant origins” can be thus activated to ensure complete DNA replication (reviewed in [30]).

**Figure 1:**
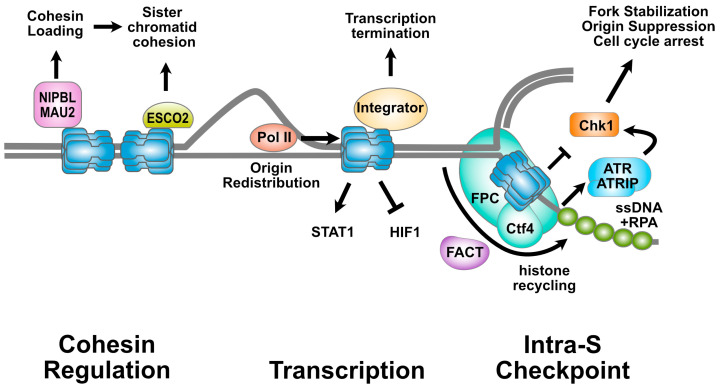
**Multiple roles of the MCM2-7 complex**. Shown is a cartoon illustrating the contributions of the MCM2-7 complex to various nuclear pathways. During DNA replication, the MCM2-7 complex forms the core of the replicative helicase. In addition to its direct role in DNA replication, the MCM2-7 complex also participates in other transactions and pathways in the nucleus. The MCM2-7 complex interacts with regulators of the cohesin complex (**left**). The MCM2-7 complex promotes recruitment of the cohesin loader (NIPBL/MAU2 heterodimer) and interacts directly with the ESCO2 cohesin acetyltransferase. MCM function also intersects with transcription (**center**). The helicase is able to displace RNA Pol II from promoters and may help unwind the DNA in advance of active polymerase. The MCM2-7 complex interacts directly with the Integrator complex, through which it controls the termination of transcription at RNA Pol II promoters. The MCM2-7 complex is essential for the intra-S phase checkpoint (**right**) and coordinates histone deposition on newly replicated DNA. Through interaction with the Fork Protection Complex (FPC), it ensures both checkpoint activation and fork stabilization when barriers are encountered. In addition, the Ctf4/AND-1 subunit of the FPC serves as a histone chaperone to help recycle histones onto the replicated DNA. The FACT complex also participates. Not drawn to scale. See text for details.

There is now also increasing evidence that MCM2-7 complexes bound to chromatin serve roles beyond DNA replication, perhaps providing one explanation for their seeming overabundance. Chromatin-bound MCM2-7 complexes serve to scaffold and integrate other activities, such as the DNA replication checkpoint response, gene transcription, and cohesin dynamics, with DNA replication. Thus, through different interacting partners, chromatin-bound MCM2-7 complexes have significant impacts on several key aspects of chromosome biology. Here, we review these non-canonical roles of the MCM helicase, summarized in Figure 1.

## 2. The MCM2-7 Complex and Cohesin Regulation

The MCM2-7 complex has significant impacts on chromosome dynamics beyond its direct role in DNA replication. This is in part due to functional interactions of the MCM2-7 complex with regulators of cohesin, a protein complex that controls chromosome compaction and segregation. Both cohesin loading and stabilization are controlled by replication licensing, albeit through distinct mechanisms.

The cohesin complex ensures proper chromosome dynamics in two mechanistically distinct ways. The tethering together of the products of DNA replication, called sister chromatids, by the cohesin complex ensures the proper alignment and accurate segregation of chromosomes, thus ensuring genome stability. Most notably in vertebrate cells, cohesin also acts in *cis* on individual chromosomes. It binds to chromatin and extrudes chromatin loops, resulting in chromosome folding and compaction. The proper formation of loops and domains, which are critically dependent on cohesin activity, are thought to ensure proper gene regulation by placing promotors and enhancers in the correct topological context (reviewed in [31]).

During the vertebrate cell cycle, the cohesin complex is largely removed from chromatin when it is phosphorylated by mitotically activated kinases, and a small fraction is protected from removal at the centromere until chromosome segregation at anaphase [32,33]. In telophase, as nuclei reform following cell division, both the MCM2-7 complex and cohesin are loaded onto chromatin [6,33]. Elegant experiments in the *Xenopus* egg extract system have shown that cohesin loading is dependent on full replication licensing [34,35]. ORC binding, Cdt1, Cdc6, and MCM loading, which together form the pre-replication complex (pre-RC), were all found to be prerequisites for cohesin loading [34,35]. When replication licensing is blocked, the cohesin complex fails to load onto chromatin. Importantly, active replication initiation, which requires the recruitment of additional proteins and results in the unwinding of the DNA helix by the MCM2-7 complex, is not a prerequisite; treatments that prevent replication initiation have no immediate impact on cohesin loading.

How does replication licensing, specifically MCM2-7 loading, control cohesin activity? Cohesin loading depends on a heterodimeric protein complex composed of the NIPBL and MAU2 proteins (Scc2 and Scc4 in *Xenopus* and budding yeast) [36,37]. This loader complex was shown to interact directly with a kinase called DDK (Dbf4-dependent kinase) [38]. DDK is also composed of two subunits—the CDC7 kinase and one of two activating subunits, either Dbf4 or Drf1 [39]. DDK interacts directly with NIPBL/MAU2, forming a soluble complex that also contains cohesin [38]. The recruitment of DDK to pre-RCs thus results in the recruitment of the cohesin loader complex, which in turn stimulates cohesin loading. The depletion of DDK from *Xenopus* egg extract prevents chromatin association of Scc2/Scc4, and kinase-inactive CDC7/Drf1 fails to recruit NIPBL/MAU2 to chromatin. The dependency on MCMs for cohesin loading onto chromatin is largely conserved in cultured somatic cells, as depletion of the essential MCM2 subunit with siRNA reduces loading of cohesin on chromatin during early S phase, but this effect was less obvious in telophase [40]. This may reflect greater temporal separation of licensing and DDK recruitment in somatic cells than in the embryonic *Xenopus* egg extract system. In addition, immunoprecipitation of the MCM2-7 complex from cultured somatic cells has been shown to co-precipitate all four core subunits of cohesin (SMC1, SMC3, Rad21, SA), but whether this was through direct interaction between the complexes is unclear [41]. Regardless of how the interaction occurs, this experiment reinforces the notion that the pre-RC has functional interactions with the cohesion machinery.

In addition to promoting cohesin loading, the MCM2-7 complex makes critical contributions to the establishment of cohesion between sister chromatids. Although most cohesin association with chromatin is dynamic throughout interphase, a small pool is stabilized during DNA replication, and this pool is thought to be critical for sister chromatid cohesion [42]. One clue came from work in budding yeast, which showed that acetylation of the SMC3 subunit of cohesin by the Eco1 acetyltransferase stabilizes its interaction with chromatin, most likely by inhibiting the ability of the Wapl (Rad61 in budding yeast) protein to remove cohesin from chromatin [43,44,45,46,47].

In a well conserved mechanism, acetylation of SMC3 during DNA replication is critical for establishing and maintaining cohesion between sister chromatids [45,46,48]. In budding yeast, the dependence on DNA replication was shown to be due to direct interaction of Eco1 with the replication processivity factor, PCNA (proliferating cell nuclear antigen), which associates with chromatin and is loaded onto primed ssDNA by RFC (replication factor C) [49]. In vertebrates, the situation is somewhat more complicated. Vertebrates express two distinct homologs of Eco1, ESCO1 and ESCO2, which share a conserved acetyltransferase domain with the budding yeast Eco1 protein but have distinct, largely disordered N-terminal extensions not found in Eco1 [50,51]. Although both enzymes modify SMC3, the establishment of cohesion between sisters during DNA replication is critically dependent on acetylation by the ESCO2 protein, while ESCO1 has broad impacts on gene expression [52,53].

In vertebrate models, the direct interaction of ESCO2 with the MCM2-7 complex is critical for sister chromatid cohesion. In *Xenopus* egg extract, the recruitment of ESCO2 (also called xEco2) to chromatin, and the resulting SMC3 acetylation, depends on assembly of the pre-RC [54,55]. The nature of the interaction between ESCO2 and the MCM2-7 complex is unusual: ESCO2 may interact with multiple MCM2-7 subunits, with a slight preference for MCM4 [56,57]. ESCO2 interacts with the MCM2-7 complex through a conserved motif in the ESCO2 N-terminal tail [55,56,58]. ESCO2 interaction with the MCM helicase is required for the establishment of sister chromatid cohesion and for acetylation of SMC3 by ESCO2 [55,58,59]. The interaction of ESCO2 with MCM may also contribute to ESCO2 stability, as derivatives that fail to interact with the MCM2-7 complex appear to be less stable [56,57,58]. The MCM-binding motif present in vertebrate ESCO2 is not conserved in budding yeast Eco1p; Eco1 may interact with MCM2 through some other mechanism [60]. Collectively, these data suggest that the establishment of sister chromatid cohesion and DNA replication are coordinated through the direct interaction between ESCO2 and the MCM2-7 complex.

In addition to its role in cohesin loading and the establishment of sister chromatid cohesion, the MCM2-7 complex impacts the activity of cohesin once it is chromatin-associated. The genome is organized into loops and topologically associated domains (TADs), in large part through the loop extrusion activity of the cohesin complex [61,62]. In an elegant study combining single-cell chromosome confirmation capture, single molecule imaging, and computer simulations, Dequeker et al. showed that the MCM2-7 complex restricts the ability of cohesin to form chromatin loops by binding chromatin and forming a physical barrier to loop extrusion, particularly in G1 cells [63]. They suggested that the cohesin complex interacts directly with MCM3 in human cells through a motif conserved in other cohesin-interacting proteins [64]. The interaction of cohesin with the MCM2-7 complex likely impacts the transcriptional landscape by affecting changes in chromosome loops and domains. This may in fact contribute to cell cycle-correlated changes in transcription between cells with bound MCM2-7 and cells in which MCM2-7 has unloaded following DNA replication or in which licensing is reduced, such as in quiescence.

## 3. Sharing the Highway: The MCM2-7 Complex and Transcription

**Figure 2:**
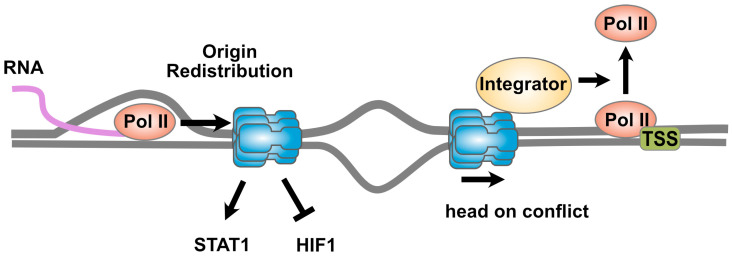
**The MCM2-7 complex and transcription.** The MCM2-7 complex is involved in mechanisms that help mitigate transcription–replication conflicts. At left, active transcription by RNA polymerase II can displace the MCM2-7 complex, thereby redistributing DNA replication origins and preventing conflicts. The Integrator complex associates with the CMG helicase and helps mitigate co-directional conflicts between the helicase at the leading edge of a replication bubble and RNA polymerase II sitting at paused promotors. The result is termination of transcription, and release of RNA polymerase II from the DNA. This allows the CMG helicase to proceed, thereby preventing activation of ATM-mediated checkpoint signaling. See text for details.

The MCM2-7 complex helps to coordinate transcription and DNA replication and mitigates their conflicts. Transcription–replication conflicts (TRCs) arise when active DNA replication and transcription on the same DNA template interfere with each other. TRCs can occur in two orientations: co-directional, in which the machineries are moving in the same direction on the template, or head-on, in which the machineries collide from opposite directions [65,66]. These conflicts can lead to replication stress and DNA double-strand breaks due to replication fork stalling and collapse. Some studies suggest that the direct interaction of the MCM2-7 complex with transcription machinery may serve to mitigate potential conflicts (see Figure 2).

The MCM2-7 complex has been copurified from cell lysates with the RNA polymerase II (RNA Pol II) holoenzyme, suggesting that these complexes interact, perhaps directly [67]. Consistent with this observation is the fact that the expression of MCM subunits is required for RNA Pol II-dependent transcription, and the MCM2-7 complex is found to localize to constitutively expressed genes [68]. This raises the possibility that the helicase may have a direct role in unwinding DNA for elongation of all RNA Pol II transcripts.

In a reciprocal manner, active transcription itself may cause redistribution of the MCM2-7 complex on the genome. The RNA Pol II complex can push MCMs on DNA, which redistributes replication origins and is likely to thereby minimize TRCs [69]. In fact, DNA replication is more likely to be initiated outside of frequently transcribed regions, suggesting a mechanism that spatially segregates transcription and DNA replication [70]. Single-molecule assays of loaded origins show that RNA Pol II can redistribute MCM2-7 on DNA fibers, and that the preinitiation complex (ORC-Cdc6-Cdt1-MCM2-7) remains intact when this happens [71]. This suggests that origin licensing remains functional, even after RNA Pol II has repositioned it. Whether this mechanism depends on specific interactions between RNA Pol II and the MCM2-7 complex has not been determined, but the redistribution of MCMs by RNA polymerase would certainly help to reduce TRCs.

In addition to helping to prevent TRCs, the MCM2-7 complex may also contribute to the resolution or prevention of a dangerous byproduct of transcription: R-loops. These three-stranded DNA:RNA hybrids occur when RNA transcripts anneal to template DNA and displace the non-template strand [72]. R-loops can present obstacles to the DNA replication machinery, leading to replication-dependent stress [72,73,74,75]. It appears the MCM2-7 complex acts as an orientation-dependent regulator of R-loop levels, helping to reduce R-loop formation in the co-directional (CD) orientation but promoting their formation in the head-on (HO) orientation, where conflicts between transcription and replication machinery are more likely to occur [74]. In fact, the MCM helicase can unwind DNA:RNA hybrids in the 3′ to 5′ orientation in vitro, suggesting a potential direct role for MCM helicase in R-loop resolution or removal [76].

In higher eukaryotes, the MCM2-7 complex minimizes transcription–replication conflicts by interacting with the Integrator complex. Integrator is a multi-subunit (INTS1–INTS15) protein complex that plays a critical role in the regulation of transcription at RNA Pol II promotors [77,78]. The interaction between the MCM2-7 complex and Integrator promotes the removal of RNA Pol II at paused promotors during replication fork progression, thus ensuring that DNA replication can proceed unimpeded [79]. The interaction between MCM2-7 and the Integrator complex occurs independently of RNA Pol II and is only observed during active DNA synthesis. Loss of Integrator function leads to an increase in co-directional conflicts and increased proximity between MCM2, MCM3, and RNA Pol II [79]. The exact nature of the interaction between Integrator and the MCM2-7 complex is not yet clear, but these data suggest a mechanism in which Integrator interaction with the MCM helicase at active replication forks prevents co-directional conflicts.

The MCM2-7 complex also plays a direct role in certain transcription pathways, such as cytokine gene activation. MCM subunits interact directly with the Stat1 transcriptional activator [80,81]. Stat1 (signal transducer and activator of transcription 1) is activated when phosphorylated by the JAK tyrosine kinase, leading to the transcription of downstream cytokine genes [82]. The phosphorylation-dependent interaction of MCM5 with Stat1 is essential for its transcriptional activity [80,81,83]. This suggests that the MCM2-7 complex can act as a chromatin-bound scaffold, enhancing certain transcriptional pathways.

The transcriptional response to hypoxia is also integrated with the function of the MCM2-7 complex, in this case in reciprocal ways. The transcription factor hypoxia-inducible factor 1 (HIF-1), which regulates the expression of genes that help mitigate oxidative damage [84,85], binds to Cdc6, thereby preventing MCM activation, though not licensing, and thus DNA replication [86,87]. In addition, HIF-1 binds directly to multiple MCM subunits, which leads to HIF-1 ubiquitination and subsequent degradation by the proteasome [86]. These data suggest that MCM2-7 complex function is downregulated during hypoxia, while HIF-1-dependent transcription may be inhibited in cells in which the MCM2-7 complex is already bound to chromatin. These mechanisms would seem to help to prevent relicensing of origins during hypoxic stress, pausing the onset of DNA replication until more favorable conditions are achieved. Given the abundance of MCM2-7 complexes present on chromatin, it is possible other transcriptional pathways, particularly those that are cell cycle related, might be found to be controlled through interaction with this complex.

## 4. MCM2-7 Complexes and the Intra-S Checkpoint

Active replication forks may encounter obstacles or lesions that cause them to stall and even collapse. Under these circumstances, the MCM2-7 complex plays an important role in the checkpoint response, both by interacting with proteins that induce the checkpoint and by maintaining active checkpoint signaling at the site of stress. The intra-S phase checkpoint is activated directly at the replication fork with the participation of the CMG helicase, while DNA double-strand breaks, both near the replication fork and elsewhere in the genome, induce a related checkpoint pathway, the DNA damage checkpoint [65,88,89]. The differences in these pathways have been reviewed elsewhere [89,90,91].

During DNA replication, the interaction of the active CMG helicase with chromatin is stabilized by a multi-subunit protein complex called the Fork Protection Complex [92]. This complex, which consists of the Timeless, TIPIN, Claspin, and AND-1 proteins (Tof1, Csm3, Mrc1, and Ctf4 in budding yeast), is recruited to the replisome following DDK-dependent phosphorylation of the MCM2-7 complex, and travels with the CMG helicase during DNA replication [93,94,95,96,97,98]. The FPC serves as a processivity factor, helping to prevent fork stalling and stabilizing forks when they do stall [99,100,101]. The FPC prevents disassembly of the replisome when barriers or nucleotide paucity cause fork stalling and is critical for fork restart once the barrier is repaired or mitigated in some way [102].

**Figure 3:**
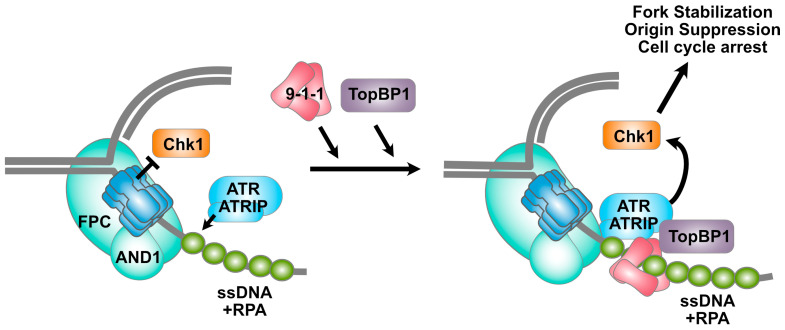
**The MCM2-7 complex and the intra-S checkpoint.** Replication barriers cause fork stalling, which in turn results in the accumulation of single-stranded DNA adjacent to the CMG helicase. The binding of RPA to the single-stranded DNA results in recruitment of the ATR kinase through its associated ATRIP protein. Subsequent loading of the Rad9-Rad1-Hus1 heterotrimeric clamp results in the recruitment of TopBP1, which activates the ATR kinase. Importantly, the TIPIN and Claspin subunits of the Fork Protection Complex (FPC) are also critical to ATR activation. The Chk1 kinase, which can interact directly with the MCM2-7 complex, is activated by ATR and mediates numerous responses to the damage signal, including fork stabilization, inhibition of origin activation, and cell cycle arrest. See text for details.

The MCM2-7 complex is critical for activation of the intra-S phase checkpoint (see Figure 3). Stalled replication forks can lead to the uncoupling of the CMG helicase from the polymerase, which in turn leads to the generation of unprotected single-stranded DNA adjacent to the stalled fork [103,104]. This ssDNA recruits the single-strand-binding complex RPA (replication protein A), which in turn recruits the Ataxia telangiectasia and Rad3-related (ATR) kinase through its ATRIP binding partner [105]. While the interaction between RPA and ATRIP is sufficient for the recruitment of ATR [106], the activation of ATR requires the synthesis of RNA primers by DNA polymerase α (Pol α) [107,108]. Polα associates with the replisome through interactions with the Ctf4/AND-1 homotrimer [109]. When replication forks stall, unprocessed primers result in ssDNA–dsDNA/RNA structures with free 5′ ends that support the loading of the 9-1-1 complex (called Rad9-Rad1-Hus1 in humans) [110]. Together, the 9-1-1 complex and Pol α recruit to the stalled replication fork the primary ATR activating protein, TopBP1 [111]. The activation of ATR at stalled replication forks is also stimulated by the FPC, whose TIPIN and Claspin subunits are critical for activation of the downstream Checkpoint Kinase 1 (Chk1) during S phase [98,112]. Chk1 in turn orchestrates many cellular responses to damage signaling, including cell cycle arrest and origin suppression. The budding yeast ortholog of Claspin, called Mrc1, has been shown to interact with the MCM6 subunit of the helicase, and mutants in which this interaction is abrogated are defective in activation of the replication checkpoint in response to certain kinds of DNA damage [113]. Thus, through the generation of ssDNA and interaction with the FPC, the CMG helicase mediates the response to fork stalling, ensuring genome maintenance in the face of DNA replication barriers.

The MCM2-7 complex itself is a direct target of both the intra-S phase checkpoint (described above) and the related DNA damage checkpoint. The DNA damage checkpoint is activated by DNA double-strand breaks, which activate the ATM (Ataxia telangiectasia mutated) kinase. This kinase is related to ATR and similarly stimulates the phosphorylation of a number of proteins to mediate cell cycle arrest and prevent origin firing [89]. MCM3 is phosphorylated by ATM in response to genotoxic stress, while MCM2 is phosphorylated by ATR [114,115]. ATR-mediated phosphorylation of MCM2 is essential for the recovery of DNA replication after activation of the checkpoint.

The MCM2-7 complex also interacts with another kinase, called Polo-like kinase (Plk1), in a mechanism that can suppress the intra-S phase checkpoint. Plk1 is a multifunctional cell cycle regulatory kinase. Plk1 interacts with the MCM2-7 complex in response to activation of the intra-S checkpoint, and there restores DNA replication [116,117]. The phosphorylation of the MCM helicase by ATM and ATR facilitates replication fork restart through an interaction with Plx1 (the *Xenopus* ortholog of Plk1). Finally, the MCM7 protein interacts directly with the ATR accessory protein called ATRIP, and cells that are hypomorphic for MCM7 expression have an intra-S phase checkpoint defect [114]. This suggests that the MCM7 subunit may play a critical role in checkpoint activation, through direct interactions with the ATR–ATRIP heterodimer.

Finally, it is interesting to note that, in the absence of DNA damage, the MCM helicase acts as a chromatin-associated sink for inactive Chk1. Chk1 binds directly to MCM helicase, and this interaction is abruptly reduced in response to DNA damage [118]. The association between Chk1 and the MCM helicase seems to serve two purposes: it promotes the rapid phosphorylation of Chk1 by locally activated ATR, and it provides a regulated mechanism for the release of phosphorylated Chk1 from chromatin—a critical step in amplifying the cell cycle checkpoint [118,119].

## 5. The MCM2-7 Complex, Histone Inheritance, and Epigenetic Memory

As the replication fork progresses along the DNA template, histones are disassembled into H2A-H2B and H3-H4 subcomplexes, and chromatin must be reassembled behind the fork. The histones deposited on the replicated strands include both the “parental” histones, including their post-translational modifications, and naïve unmarked histones. The MCM2-7 complex makes critical contributions to chromatin reassembly during DNA replication. Through direct interaction with the MCM2 subunit of the helicase, the AND-1 (Ctf4 in budding yeast) subunits of the FPC chaperone the parental (H3–H4)_2_ tetramers onto the newly formed DNA behind the fork. This mechanism ensures the inheritance of certain histone marks on the lagging strand, and this is critically important for certain types of epigenetic inheritance (reviewed in [120]). The disruption of the interaction with AND-1/Ctf4 results in asymmetric inheritance of chromatin marks and changes in cell identity [121,122,123,124,125]. There is also evidence in yeast that the FACT (Facilitator of Chromatin Transcription) histone chaperone promotes histone recycling through interaction with the MCM helicase [126,127,128,129]. In budding yeast, FACT interacts with the MCM2 subunit, while, in higher eukaryotes, FACT may have a different interacting partner within the complex [126,127,129,130,131]. Indeed, the FACT complex, originally characterized for its role in transcription, seems to play critical roles in assuring the efficiency of DNA replication through different kinds of chromatin, suggesting that the coupling of DNA replication with histone repositioning improves the efficiency of DNA replication [128,129,131,132,133]. Collectively, the data indicate that the MCM2-7 complex at the core of the active replication helicase coordinates histone deposition adjacent to the replication fork. Interactions between the MCM2-7 complex and histone chaperones play critical roles during DNA replication in the local recycling of histones behind the fork, helping to maintain chromatin marks and cell identity through genome duplication.

## 6. Conclusions

We have seen in this review that the MCM2-7 complex plays many roles beyond the direct unwinding of the DNA double helix during DNA replication. The MCM2-7 complex serves as a hub, playing essential roles in cohesin regulation, genome organization, and the coordination of transcription with other DNA transactions on the crowded genome. Through both direct physical and functional interactions, the MCM2-7 complex helps coordinate DNA replication with other pathways required for cellular homeostasis, histone deposition, stress responses, and recovery from damage. Given these observations, the MCM2-7 complex can be thought of as a central player in the maintenance of genome integrity.

## References

[1] Remus D., Beuron F., Tolun G., Griffith J.D., Morris E.P., Diffley J.F. (2009). Concerted Loading of Mcm2–7 Double Hexamers around DNA during DNA Replication Origin Licensing. Cell.

[2] Davey M.J., Indiani C., O’Donnell M. (2003). Reconstitution of the Mcm2-7p Heterohexamer, Subunit Arrangement, and ATP Site Architecture. J. Biol. Chem..

[3] Li N., Zhai Y., Zhang Y., Li W., Yang M., Lei J., Tye B.-K., Gao N. (2015). Structure of the eukaryotic MCM complex at 3.8 Å. Nature.

[4] Bochman M.L., Schwacha A. (2008). The Mcm2-7 Complex Has In Vitro Helicase Activity. Mol. Cell.

[5] Ishimi Y. (1997). A DNA Helicase Activity Is Associated with an MCM4, -6, and -7 Protein Complex. J. Biol. Chem..

[6] Evrin C., Clarke P., Zech J., Lurz R., Sun J., Uhle S., Li H., Stillman B., Speck C. (2009). A double-hexameric MCM2-7 complex is loaded onto origin DNA during licensing of eukaryotic DNA replication. Proc. Natl. Acad. Sci. USA.

[7] Bell S.P., Stillman B. (1992). ATP-dependent recognition of eukaryotic origins of DNA replication by a multiprotein complex. Nature.

[8] Li N., Lam W.H., Zhai Y., Cheng J., Cheng E., Zhao Y., Gao N., Tye B.-K. (2018). Structure of the origin recognition complex bound to DNA replication origin. Nature.

[9] McGarry T.J., Kirschner M.W. (1998). Geminin, an Inhibitor of DNA Replication, Is Degraded during Mitosis. Cell.

[10] Méndez J., Stillman B. (2000). Chromatin Association of Human Origin Recognition Complex, Cdc6, and Minichromosome Maintenance Proteins during the Cell Cycle: Assembly of Prereplication Complexes in Late Mitosis. Mol. Cell. Biol..

[11] Donovan S., Harwood J., Drury L.S., Diffley J.F.X. (1997). Cdc6p-dependent loading of Mcm proteins onto pre-replicative chromatin in budding yeast. Proc. Natl. Acad. Sci. USA.

[12] Maiorano D., Moreau J., Méchali M. (2000). XCDT1 is required for the assembly of pre-replicative complexes in Xenopus laevis. Nature.

[13] Nishitani H., Lygerou Z., Nishimoto T., Nurse P. (2000). The Cdt1 protein is required to license DNA for replication in fission yeast. Nature.

[14] Lei M., Tye B.K. (2001). Initiating DNA synthesis: From recruiting to activating the MCM complex. J. Cell Sci..

[15] Cheng J., Li N., Huo Y., Dang S., Tye B.-K., Gao N., Zhai Y. (2022). Structural Insight into the MCM double hexamer activation by Dbf4-Cdc7 kinase. Nat. Commun..

[16] Li N., Gao N., Zhai Y. (2023). DDK promotes DNA replication initiation: Mechanistic and structural insights. Curr. Opin. Struct. Biol..

[17] Sheu Y.-J., Stillman B. (2006). Cdc7-Dbf4 Phosphorylates MCM Proteins via a Docking Site-Mediated Mechanism to Promote S Phase Progression. Mol. Cell.

[18] Douglas M.E., Ali F.A., Costa A., Diffley J.F.X. (2018). The mechanism of eukaryotic CMG helicase activation. Nature.

[19] Gambus A., Jones R.C., Sanchez-Diaz A., Kanemaki M., van Deursen F., Edmondson R.D., Labib K. (2006). GINS maintains association of Cdc45 with MCM in replisome progression complexes at eukaryotic DNA replication forks. Nat. Cell Biol..

[20] Yuan Z., Georgescu R., Bai L., Zhang D., Li H., O’donnell M.E. (2020). DNA unwinding mechanism of a eukaryotic replicative CMG helicase. Nat. Commun..

[21] Bailey R., Moreno S.P., Gambus A. (2015). Termination of DNA replication forks: “Breaking up is hard to do”. Nucleus.

[22] Dewar J.M., Low E., Mann M., Räschle M., Walter J.C. (2017). CRL2^Lrr1^ promotes unloading of the vertebrate replisome from chromatin during replication termination. Genes Dev..

[23] Maric M., Maculins T., De Piccoli G., Labib K. (2014). Cdc48 and a ubiquitin ligase drive disassembly of the CMG helicase at the end of DNA replication. Science.

[24] Moreno S.P., Bailey R., Campion N., Herron S., Gambus A. (2014). Polyubiquitylation drives replisome disassembly at the termination of DNA replication. Science.

[25] Jagannathan M., Nguyen T., Gallo D., Luthra N., Brown G.W., Saridakis V., Frappier L. (2014). A Role for USP7 in DNA Replication. Mol. Cell. Biol..

[26] Nishiyama A., Frappier L., Méchali M. (2011). MCM-BP regulates unloading of the MCM2–7 helicase in late S phase. Genes Dev..

[27] Kusunoki S., Ishimi Y. (2014). Interaction of human minichromosome maintenance protein-binding protein with minichromosome maintenance 2–7. FEBS J..

[28] Ge X.Q., Jackson D.A., Blow J.J. (2007). Dormant origins licensed by excess Mcm2–7 are required for human cells to survive replicative stress. Genes Dev..

[29] Ibarra A., Schwob E., Méndez J. (2008). Excess MCM proteins protect human cells from replicative stress by licensing backup origins of replication. Proc. Natl. Acad. Sci. USA.

[30] Courtot L., Hoffmann J.-S., Bergoglio V. (2018). The Protective Role of Dormant Origins in Response to Replicative Stress. Int. J. Mol. Sci..

[31] Agarwal A., Korsak S., Choudhury A., Plewczynski D. (2023). The dynamic role of cohesin in maintaining human genome architecture. BioEssays.

[32] Sumara I., Vorlaufer E., Stukenberg P., Kelm O., Redemann N., Nigg E.A., Peters J.-M. (2002). The Dissociation of Cohesin from Chromosomes in Prophase Is Regulated by Polo-like Kinase. Mol. Cell.

[33] Sumara I., Vorlaufer E., Gieffers C., Peters B.H., Peters J.-M. (2000). Characterization of Vertebrate Cohesin Complexes and Their Regulation in Prophase. J. Cell Biol..

[34] Takahashi T.S., Yiu P., Chou M.F., Gygi S., Walter J.C. (2004). Recruitment of Xenopus Scc2 and cohesin to chromatin requires the pre-replication complex. Nat. Cell Biol..

[35] Gillespie P.J., Hirano T. (2004). Scc2 Couples Replication Licensing to Sister Chromatid Cohesion in Xenopus Egg Extracts. Curr. Biol..

[36] Ciosk R., Shirayama M., Shevchenko A., Tanaka T., Toth A., Shevchenko A., Nasmyth K. (2000). Cohesin’s Binding to Chromosomes Depends on a Separate Complex Consisting of Scc2 and Scc4 Proteins. Mol. Cell.

[37] Watrin E., Schleiffer A., Tanaka K., Eisenhaber F., Nasmyth K., Peters J.-M. (2006). Human Scc4 is required for cohesin binding to chromatin, sister-chromatid cohesion, and mitotic progression. Curr. Biol..

[38] Takahashi T.S., Basu A., Bermudez V., Hurwitz J., Walter J.C. (2008). Cdc7–Drf1 kinase links chromosome cohesion to the initiation of DNA replication in *Xenopus* egg extracts. Genes Dev..

[39] Takahashi T.S., Walter J.C. (2005). Cdc7–Drf1 is a developmentally regulated protein kinase required for the initiation of vertebrate DNA replication. Gene Dev..

[40] Zheng G., Kanchwala M., Xing C., Yu H., States U., University of Texas Southwestern Medical Center (2018). MCM2–7-dependent cohesin loading during S phase promotes sister-chromatid cohesion. eLife.

[41] Guillou E., Ibarra A., Coulon V., Casado-Vela J., Rico D., Casal I., Schwob E., Losada A., Méndez J. (2010). Cohesin organizes chromatin loops at DNA replication factories. Genes Dev..

[42] Gerlich D., Koch B., Dupeux F., Peters J.-M., Ellenberg J. (2006). Live-Cell Imaging Reveals a Stable Cohesin-Chromatin Interaction after but Not before DNA Replication. Curr. Biol..

[43] Skibbens R.V., Corson L.B., Koshland D., Hieter P. (1999). Ctf7p is essential for sister chromatid cohesion and links mitotic chromosome structure to the DNA replication machinery. Genes Dev..

[44] Tóth A., Ciosk R., Uhlmann F., Galova M., Schleiffer A., Nasmyth K. (1999). Yeast Cohesin complex requires a conserved protein, Eco1p(Ctf7), to establish cohesion between sister chromatids during DNA replication. Genes Dev..

[45] Zhang J., Shi X., Li Y., Kim B.-J., Jia J., Huang Z., Yang T., Fu X., Jung S.Y., Wang Y. (2008). Acetylation of Smc3 by Eco1 Is Required for S Phase Sister Chromatid Cohesion in Both Human and Yeast. Mol. Cell.

[46] Ünal E., Heidinger-Pauli J.M., Kim W., Guacci V., Onn I., Gygi S.P., Koshland D.E. (2008). A Molecular Determinant for the Establishment of Sister Chromatid Cohesion. Science.

[47] Ben-Shahar T.R., Heeger S., Lehane C., East P., Flynn H., Skehel M., Uhlmann F. (2008). Eco1-Dependent Cohesin Acetylation During Establishment of Sister Chromatid Cohesion. Science.

[48] Uhlmann F., Nasmyth K. (1998). Cohesion between sister chromatids must be established during DNA replication. Curr. Biol..

[49] Moldovan G.-L., Pfander B., Jentsch S. (2006). PCNA Controls Establishment of Sister Chromatid Cohesion during S Phase. Mol. Cell.

[50] Hou F., Zou H. (2005). Two Human Orthologues of Eco1/Ctf7 Acetyltransferases Are Both Required for Proper Sister-Chromatid Cohesion. Mol. Biol. Cell.

[51] Bellows A.M., Kenna M.A., Cassimeris L., Skibbens R.V. (2003). Human EFO1p exhibits acetyltransferase activity and is a unique combination of linker histone and Ctf7p/Eco1p chromatid cohesion establishment domains. Nucleic Acids Res..

[52] Alomer R.M., da Silva E.M.L., Chen J., Piekarz K.M., McDonald K., Sansam C.G., Sansam C.L., Rankin S. (2017). Esco1 and Esco2 regulate distinct cohesin functions during cell cycle progression. Proc. Natl. Acad. Sci. USA.

[53] Rahman S., Jones M.J.K., Jallepalli P.V. (2015). Cohesin recruits the Esco1 acetyltransferase genome wide to repress transcription and promote cohesion in somatic cells. Proc. Natl. Acad. Sci. USA.

[54] Lafont A.L., Song J., Rankin S. (2010). Sororin cooperates with the acetyltransferase Eco2 to ensure DNA replication-dependent sister chromatid cohesion. Proc. Natl. Acad. Sci. USA.

[55] Higashi T.L., Ikeda M., Tanaka H., Nakagawa T., Bando M., Shirahige K., Kubota Y., Takisawa H., Masukata H., Takahashi T.S. (2012). The Prereplication Complex Recruits XEco2 to Chromatin to Promote Cohesin Acetylation in Xenopus Egg Extracts. Curr. Biol..

[56] Ivanov M.P., Ladurner R., Poser I., Beveridge R., Rampler E., Hudecz O., Novatchkova M., Hériché J., Wutz G., van der Lelij P. (2018). The replicative helicase MCM recruits cohesin acetyltransferase ESCO2 to mediate centromeric sister chromatid cohesion. EMBO J..

[57] Minamino M., Tei S., Negishi L., Kanemaki M.T., Yoshimura A., Sutani T., Bando M., Shirahige K. (2018). Temporal Regulation of ESCO2 Degradation by the MCM Complex, the CUL4-DDB1-VPRBP Complex, and the Anaphase-Promoting Complex. Curr. Biol..

[58] Bender D., Da Silva E.M.L., Chen J., Poss A., Gawey L., Rulon Z., Rankin S. (2020). Multivalent interaction of ESCO2 with the replication machinery is required for sister chromatid cohesion in vertebrates. Proc. Natl. Acad. Sci. USA.

[59] Song J., Lafont A., Chen J., Wu F.M., Shirahige K., Rankin S. (2012). Cohesin acetylation promotes sister chromatid cohesion only in association with the replication machin-ery. J. Biol. Chem..

[60] Yoshimura A., Sutani T., Shirahige K. (2021). Functional control of Eco1 through the MCM complex in sister chromatid cohesion. Gene.

[61] Rao S.S.P., Huang S.-C., St Hilaire B.G., Engreitz J.M., Perez E.M., Kieffer-Kwon K.-R., Sanborn A.L., Johnstone S.E., Bascom G.D., Bochkov I.D. (2017). Cohesin Loss Eliminates All Loop Domains. Cell.

[62] Davidson I.F., Bauer B., Goetz D., Tang W., Wutz G., Peters J.-M. (2019). DNA loop extrusion by human cohesin. Science.

[63] Dequeker B.J.H., Scherr M.J., Brandão H.B., Gassler J., Powell S., Gaspar I., Flyamer I.M., Lalic A., Tang W., Stocsits R. (2022). MCM complexes are barriers that restrict cohesin-mediated loop extrusion. Nature.

[64] Li Y., Haarhuis J.H.I., Cacciatore Á.S., Oldenkamp R., Van Ruiten M.S., Willems L., Teunissen H., Muir K.W., De Wit E., Rowland B.D. (2020). The structural basis for cohesin–CTCF-anchored loops. Nature.

[65] García-Muse T., Aguilera A. (2016). Transcription–replication conflicts: How they occur and how they are resolved. Nat. Rev. Mol. Cell Biol..

[66] Hamperl S., Cimprich K.A. (2016). Conflict Resolution in the Genome: How Transcription and Replication Make It Work. Cell.

[67] Yankulov K., Todorov I., Romanowski P., Licatalosi D., Cilli K., McCracken S., Laskey R., Bentley D.L. (1999). MCM Proteins Are Associated with RNA Polymerase II Holoenzyme. Mol. Cell. Biol..

[68] Snyder M., Huang X.-Y., Zhang J.J. (2009). The minichromosome maintenance proteins 2-7 (MCM2-7) are necessary for RNA polymerase II (Pol II)-mediated transcription. J. Biol. Chem..

[69] Gros J., Kumar C., Lynch G., Yadav T., Whitehouse I., Remus D. (2015). Post-licensing Specification of Eukaryotic Replication Origins by Facilitated Mcm2-7 Sliding along DNA. Mol. Cell.

[70] Liu Y., Ai C., Gan T., Wu J., Jiang Y., Liu X., Lu R., Gao N., Li Q., Ji X. (2021). Transcription shapes DNA replication initiation to preserve genome integrity. Genome Biol..

[71] Scherr M.J., Wahab S.A., Remus D., Duderstadt K.E. (2022). Mobile origin-licensing factors confer resistance to conflicts with RNA polymerase. Cell Rep..

[72] Petermann E., Lan L., Zou L. (2022). Sources, resolution and physiological relevance of R-loops and RNA–DNA hybrids. Nat. Rev. Mol. Cell Biol..

[73] Gan W., Guan Z., Liu J., Gui T., Shen K., Manley J.L., Li X. (2011). R-loop-mediated genomic instability is caused by impairment of replication fork progression. Genes Dev..

[74] Hamperl S., Bocek M.J., Saldivar J.C., Swigut T., Cimprich K.A. (2017). Transcription-Replication Conflict Orientation Modulates R-Loop Levels and Activates Distinct DNA Damage Responses. Cell.

[75] Aguilera A., Gómez-González B. (2017). DNA–RNA hybrids: The risks of DNA breakage during transcription. Nat. Struct. Mol. Biol..

[76] Shin J.-H., Kelman Z. (2006). The Replicative Helicases of Bacteria, Archaea, and Eukarya Can Unwind RNA-DNA Hybrid Substrates. J. Biol. Chem..

[77] Baillat D., Hakimi M.-A., Näär A.M., Shilatifard A., Cooch N., Shiekhattar R. (2005). Integrator, a Multiprotein Mediator of Small Nuclear RNA Processing, Associates with the C-Terminal Repeat of RNA Polymerase II. Cell.

[78] Stein C.B., Field A.R., Mimoso C.A., Zhao C., Huang K.-L., Wagner E.J., Adelman K. (2022). Integrator endonuclease drives promoter-proximal termination at all RNA polymerase II-transcribed loci. Mol. Cell.

[79] Bhowmick R., Mehta K.P., Lerdrup M., Cortez D. (2023). Integrator facilitates RNAPII removal to prevent transcription-replication collisions and genome instability. Mol. Cell.

[80] Zhang J.J., Zhao Y., Chait B.T., Lathem W.W., Ritzi M., Knippers R., Darnell J.E. (1998). Ser727-dependent recruitment of MCM5 by Stat1α in IFN-γ-induced transcriptional activation. EMBO J..

[81] DaFonseca C.J., Shu F., Zhang J.J. (2001). Identification of two residues in MCM5 critical for the assembly of MCM complexes and Stat1-mediated transcription activation in response to IFN-γ. Proc. Natl. Acad. Sci. USA.

[82] Wen Z., Zhong Z., Darnell J.E. (1995). Maximal activation of transcription by statl and stat3 requires both tyrosine and serine phosphorylation. Cell.

[83] Snyder M., He W., Zhang J.J. (2005). The DNA replication factor MCM5 is essential for Stat1-mediated transcriptional activation. Proc. Natl. Acad. Sci. USA.

[84] Wang G.L., Jiang B.-H., Rue E.A., Semenza G.L. (1995). Hypoxia-inducible factor 1 is a basic-helix-loop-helix-PAS heterodimer regulated by cellular O_2_ tension. Proc. Natl. Acad. Sci. USA.

[85] Iyer N.V., Kotch L.E., Agani F., Leung S.W., Laughner E., Wenger R.H., Gassmann M., Gearhart J.D., Lawler A.M., Yu A.Y. (1998). Cellular and developmental control of O_2_ homeostasis by hypoxia-inducible factor 1α. Genes Dev..

[86] Hubbi M.E., Luo W., Baek J.H., Semenza G.L. (2011). MCM Proteins Are Negative Regulators of Hypoxia-Inducible Factor 1. Mol. Cell.

[87] Hubbi M.E., Kshitiz K., Gilkes D.M., Rey S., Wong C.C., Luo W., Kim D.-H., Dang C.V., Levchenko A., Semenza G.L. (2013). A Nontranscriptional Role for HIF-1α as a Direct Inhibitor of DNA Replication. Sci. Signal..

[88] Katou Y., Kanoh Y., Bando M., Noguchi H., Tanaka H., Ashikari T., Sugimoto K., Shirahige K. (2003). S-phase checkpoint proteins Tof1 and Mrc1 form a stable replication-pausing complex. Nature.

[89] Waterman D.P., Haber J.E., Smolka M.B. (2020). Checkpoint Responses to DNA Double-Strand Breaks. Annu. Rev. Biochem..

[90] Galanti L., Pfander B. (2018). Right time, right place— DNA damage and DNA replication checkpoints collectively safeguard S phase. EMBO J..

[91] Branzei D., Foiani M. (2007). Interplay of replication checkpoints and repair proteins at stalled replication forks. DNA Repair.

[92] Numata Y., Ishihara S., Hasegawa N., Nozaki N., Ishimi Y. (2010). Interaction of human MCM2-7 proteins with TIM, TIPIN and Rb. J. Biochem..

[93] Chao W.C., Murayama Y., Muñoz S., Costa A., Uhlmann F., Singleton M.R. (2015). Structural Studies Reveal the Functional Modularity of the Scc2-Scc4 Cohesin Loader. Cell Rep..

[94] Bando M., Katou Y., Komata M., Tanaka H., Itoh T., Sutani T., Shirahige K. (2009). Csm3, Tof1, and Mrc1 Form a Heterotrimeric Mediator Complex That Associates with DNA Replication Forks. J. Biol. Chem..

[95] Bastia D., Srivastava P., Zaman S., Choudhury M., Mohanty B.K., Bacal J., Langston L.D., Pasero P., O’donnell M.E. (2016). Phosphorylation of CMG helicase and Tof1 is required for programmed fork arrest. Proc. Natl. Acad. Sci. USA.

[96] Baretić D., Jenkyn-Bedford M., Aria V., Cannone G., Skehel M., Yeeles J.T. (2020). Cryo-EM Structure of the Fork Protection Complex Bound to CMG at a Replication Fork. Mol. Cell.

[97] Gambus A., van Deursen F., Polychronopoulos D., Foltman M., Jones R.C., Edmondson R.D., Calzada A., Labib K. (2009). A key role for Ctf4 in coupling the MCM2-7 helicase to DNA polymerase α within the eukaryotic replisome. EMBO J..

[98] Liu S., Bekker-Jensen S., Mailand N., Lukas C., Bartek J., Lukas J. (2006). Claspin Operates Downstream of TopBP1 To Direct ATR Signaling towards Chk1 Activation. Mol. Cell. Biol..

[99] Tourrière H., Versini G., Cordón-Preciado V., Alabert C., Pasero P. (2005). Mrc1 and Tof1 Promote Replication Fork Progression and Recovery Independently of Rad53. Mol. Cell.

[100] Ünsal-Kaçmaz K., Chastain P.D., Qu P.-P., Minoo P., Cordeiro-Stone M., Sancar A., Kaufmann W.K. (2007). The Human Tim/Tipin Complex Coordinates an Intra-S Checkpoint Response to UV That Slows Replication Fork Displacement. Mol. Cell. Biol..

[101] Leman A.R., Noguchi C., Lee C.Y., Noguchi E. (2010). Human Timeless and Tipin stabilize replication forks and facilitate sister-chromatid cohesion. J. Cell Sci..

[102] Calzada A., Hodgson B., Kanemaki M., Bueno A., Labib K. (2005). Molecular anatomy and regulation of a stable replisome at a paused eukaryotic DNA replication fork. Genes Dev..

[103] Byun T.S., Pacek M., Yee M.-C., Walter J.C., Cimprich K.A. (2005). Functional uncoupling of MCM helicase and DNA polymerase activities activates the ATR-dependent checkpoint. Genes Dev..

[104] Nedelcheva M.N., Roguev A., Dolapchiev L.B., Shevchenko A., Taskov H.B., Shevchenko A., Stewart A.F., Stoynov S.S. (2005). Uncoupling of Unwinding from DNA Synthesis Implies Regulation of MCM Helicase by Tof1/Mrc1/Csm3 Checkpoint Complex. J. Mol. Biol..

[105] Zou L., Elledge S.J. (2003). Sensing DNA Damage Through ATRIP Recognition of RPA-ssDNA Complexes. Science.

[106] MacDougall C.A., Byun T.S., Van C., Yee M.-C., Cimprich K.A. (2007). The structural determinants of checkpoint activation. Genes Dev..

[107] Michael W.M., Ott R., Fanning E., Newport J. (2000). Activation of the DNA Replication Checkpoint Through RNA Synthesis by Primase. Science.

[108] Van C., Yan S., Michael W.M., Waga S., Cimprich K.A. (2010). Continued primer synthesis at stalled replication forks contributes to checkpoint activation. J. Cell Biol..

[109] Simon A.C., Zhou J.C., Perera R.L., van Deursen F., Evrin C., Ivanova M.E., Kilkenny M.L., Renault L., Kjaer S., Matak-Vinković D. (2014). A Ctf4 trimer couples the CMG helicase to DNA polymerase α in the eukaryotic replisome. Nature.

[110] Castaneda J.C., Schrecker M., Remus D., Hite R.K. (2022). Mechanisms of loading and release of the 9-1-1 checkpoint clamp. Nat. Struct. Mol. Biol..

[111] Mordes D.A., Glick G.G., Zhao R., Cortez D. (2008). TopBP1 activates ATR through ATRIP and a PIKK regulatory domain. Genes Dev..

[112] Kemp M.G., Akan Z., Yilmaz S., Grillo M., Smith-Roe S.L., Kang T.-H., Cordeiro-Stone M., Kaufmann W.K., Abraham R.T., Sancar A. (2010). Tipin-Replication Protein A Interaction Mediates Chk1 Phosphorylation by ATR in Response to Genotoxic Stress. J. Biol. Chem..

[113] Komata M., Bando M., Araki H., Shirahige K. (2009). The Direct Binding of Mrc1, a Checkpoint Mediator, to Mcm6, a Replication Helicase, Is Essential for the Replication Checkpoint against Methyl Methanesulfonate-Induced Stress. Mol. Cell. Biol..

[114] Cortez D., Glick G., Elledge S.J. (2004). Minichromosome maintenance proteins are direct targets of the ATM and ATR checkpoint kinases. Proc. Natl. Acad. Sci. USA.

[115] Yoo H.Y., Shevchenko A., Shevchenko A., Dunphy W.G. (2004). Mcm2 Is a Direct Substrate of ATM and ATR during DNA Damage and DNA Replication Checkpoint Responses. J. Biol. Chem..

[116] Trenz K., Errico A., Costanzo V. (2008). Plx1 is required for chromosomal DNA replication under stressful conditions. EMBO J..

[117] Tsvetkov L., Stern D.F. (2005). Interaction of Chromatin-associated Plk1 and Mcm7. J. Biol. Chem..

[118] Han X., Aslanian A., Fu K., Tsuji T., Zhang Y. (2014). The Interaction between Checkpoint Kinase 1 (Chk1) and the Minichromosome Maintenance (MCM) Complex Is Required for DNA Damage-induced Chk1 Phosphorylation. J. Biol. Chem..

[119] Han X., Pozo F.M., Wisotsky J.N., Wang B., Jacobberger J.W., Zhang Y. (2015). Phosphorylation of Minichromosome Maintenance 3 (MCM3) by Checkpoint Kinase 1 (Chk1) Negatively Regulates DNA Replication and Checkpoint Activation. J. Biol. Chem..

[120] Escobar T.M., Loyola A., Reinberg D. (2021). Parental nucleosome segregation and the inheritance of cellular identity. Nat. Rev. Genet..

[121] Petryk N., Dalby M., Wenger A., Stromme C.B., Strandsby A., Andersson R., Groth A. (2018). MCM2 promotes symmetric inheritance of modified histones during DNA replication. Science.

[122] Wenger A., Biran A., Alcaraz N., Redó-Riveiro A., Sell A.C., Krautz R., Flury V., Reverón-Gómez N., Solis-Mezarino V., Völker-Albert M. (2023). Symmetric inheritance of parental histones governs epigenome maintenance and embryonic stem cell identity. Nat. Genet..

[123] Gan H., Serra-Cardona A., Hua X., Zhou H., Labib K., Yu C., Zhang Z. (2018). The Mcm2-Ctf4-Polα Axis Facilitates Parental Histone H3-H4 Transfer to Lagging Strands. Mol. Cell.

[124] Li Z., Hua X., Serra-Cardona A., Xu X., Gan S., Zhou H., Yang W.-S., Chen C.-L., Xu R.-M., Zhang Z. (2020). DNA polymerase α interacts with H3-H4 and facilitates the transfer of parental histones to lagging strands. Sci. Adv..

[125] Kang Y.-H., Farina A., Bermudez V.P., Tappin I., Du F., Galal W.C., Hurwitz J. (2013). Interaction between human Ctf4 and the Cdc45/Mcm2-7/GINS (CMG) replicative helicase. Proc. Natl. Acad. Sci. USA.

[126] Foltman M., Evrin C., De Piccoli G., Jones R.C., Edmondson R.D., Katou Y., Nakato R., Shirahige K., Labib K. (2013). Eukaryotic Replisome Components Cooperate to Process Histones During Chromosome Replication. Cell Rep..

[127] Wang X., Tang Y., Xu J., Leng H., Shi G., Hu Z., Wu J., Xiu Y., Feng J., Li Q. (2023). The N-terminus of Spt16 anchors FACT to MCM2–7 for parental histone recycling. Nucleic Acids Res..

[128] Yang J., Zhang X., Feng J., Leng H., Li S., Xiao J., Liu S., Xu Z., Xu J., Li D. (2016). The Histone Chaperone FACT Contributes to DNA Replication-Coupled Nucleosome Assembly. Cell Rep..

[129] Safaric B., Chacin E., Scherr M.J., Rajappa L., Gebhardt C., Kurat C.F., Cordes T.E., Duderstadt K. (2022). The fork protection complex recruits FACT to reorganize nucleosomes during replication. Nucleic Acids Res..

[130] Tan B.C., Liu H., Lin C.-L., Lee S.-C. (2010). Functional cooperation between FACT and MCM is coordinated with cell cycle and differential complex formation. J. Biomed. Sci..

[131] Tan B.C.-M., Chien C.-T., Hirose S., Lee S.-C. (2006). Functional cooperation between FACT and MCM helicase facilitates initiation of chromatin DNA replication. EMBO J..

[132] Nathanailidou P., Dhakshnamoorthy J., Xiao H., Zofall M., Holla S., O’neill M., Andresson T., Wheeler D., Grewal S.I.S. (2024). Specialized replication of heterochromatin domains ensures self-templated chromatin assembly and epigenetic inheritance. Proc. Natl. Acad. Sci. USA.

[133] Miller C.L., Winston F. (2023). The conserved histone chaperone Spt6 is strongly required for DNA replication and genome stability. Cell Rep..

